# An Integrated Approach to Assess Exposure and Health-Risk from Polycyclic Aromatic Hydrocarbons (PAHs) in a Fastener Manufacturing Industry

**DOI:** 10.3390/ijerph110909578

**Published:** 2014-09-15

**Authors:** Hsin-I Hsu, Ming-Yeng Lin, Yu-Cheng Chen, Wang-Yi Chen, Chungsik Yoon, Mei-Ru Chen, Perng-Jy Tsai

**Affiliations:** 1 Department of Environmental and Occupational Health, Medical College, National Cheng Kung University, 138, Sheng-Li Rd., Tainan 70428, Taiwan; E-Mails: scenicii@gmail.com (H.-I.H.); m_lin@mail.ncku.edu.tw (M.-Y.L.); chenwy@mail.iosh.gov.tw (W.-I.C.); 2 Division of Environmental Health and Occupational Medicine, National Health Research Institutes, 35 Keyan Road, Zhunan Town, Miaoli County 350, Taiwan; E-Mail: yucheng@nhri.org.tw; 3 Department of Environmental Health, Seoul National University, 1 Gwanak-ro, Gwanak-gu, Seoul 151-742, Korea; E-Mail: csyoon@snu.ac.kr; 4 Department of Occupational Safety and Health, Chung Hwa University of Medical Technology, Wenhwa 1st St., Rende Dist., Tainan City 71703, Taiwan

**Keywords:** polycyclic aromatic hydrocarbons, oil mist, exposure assessment, health-risk assessment, metal work fluid

## Abstract

An integrated approach was developed to assess exposure and health-risk from polycyclic aromatic hydrocarbons (PAHs) contained in oil mists in a fastener manufacturing industry. One previously developed model and one new model were adopted for predicting oil mist exposure concentrations emitted from metal work fluid (MWF) and PAHs contained in MWF by using the fastener production rate (*Pr*) and cumulative fastener production rate (*CPr*) as predictors, respectively. By applying the annual *Pr* and *CPr* records to the above two models, long-term workplace PAH exposure concentrations were predicted. In addition, true exposure data was also collected from the field. The predicted and measured concentrations respectively served as the prior and likelihood distributions in the Bayesian decision analysis (BDA), and the resultant posterior distributions were used to determine the long-term exposure and health-risks posed on workers. Results show that long term exposures to PAHs would result in a 3.1%, 96.7%, and 73.4% chance of exceeding the PEL-TWA (0.2 mg/m^3^), action level (0.1 mg/m^3^), and acceptable health risk (10^−3^), respectively. In conclusion, preventive measures should be taken immediately to reduce workers’ PAH exposures.

## 1. Introduction 

To conduct exposure assessments for workers exposed to chemicals with chronic health effects, workers’ long term exposure data must be collected from the field. As a result, many measured data are needed from different periods leading to a massive burden on both manpower and cost for the industry involved. On the other hand, if only a small amount of field sampling data were available for a similar exposure group (SEG), the Bayesian decision analysis (BDA) can be chosen as an approach for determining the exposure profile [1,2,3,4,5]. In principle, BDA can provide a transparent method for incorporating the relative certainty of the information or data used to produce a judgment probability chart [3,6]. The use of BDA requires knowledge of both prior and likelihood exposure distributions for the targeted SEG. The resultant posterior exposure distribution can be used to describe its exposure profile [3]. In theory, the limited measured concentrations can be used to determine the likelihood exposure distribution in BDA. For determining the prior exposure distribution, many methodologies have been adopted by industrial hygienists, such as the expert systems [1,2,7,8], numerical models [4,8,9], and surrogate exposure methods [3,8,10]. It should be noted that the use of an expert system might lead to inaccurate estimations of the posterior distribution due to inherent large variations among experts [1,2,7,8]. If the numerical model is adopted, a lot of environmental and workforce information suitable to establish the boundary conditions of the numerical method are needed [11]. As for the surrogate method, confirmation of the effectiveness of the surrogate in predicting the exposures of interest is required. 

According to Taiwan’s governmental statistics in 2012, there were ~1300 fastener manufacturing industries, employing ~24,000 workers. The annual production rate was ~1,380,000 tons/year accounting for ~16% of the global production. Seven industrial processes, including wire drawing, forming, threading, cleaning, heat treatment, surface treatment, and packaging/shipping, are involved in the industry. Among them, mineral oil-based metalworking fluids (MWFs) are used in the forming, threading, and heat treatment processes for cooling, lubricating, and corrosion inhibition purposes. As a result, workers can be exposed to oil mists due to the use of these MWFs in the manufacturing processes [12,13]. Given that mineral oils are produced from petroleum distillates, MWFs and the emitted oil mists are likely to contain polycyclic aromatic hydrocarbons (PAHs). Epidemiological and animal studies have indicated that long-term exposures to PAHs might result in increased lung cancer rates [14,15,16,17], although it should be noted that the related research was conducted only on a cross-sectional basis due to both cost and manpower constraints [18,19,20]. The lack of long-term exposure data would lead to inadequacy in assessing exposure and health-risk for fastener manufacturing industry workers.

In this study, we developed a predictive model for PAHs contained in MWFs, and then combined it with a previously developed oil mist exposure concentration predictive model [21] for predicting PAH exposure concentrations of fastener manufacturing workers. The predicted concentrations and field measured PAH concentrations then served as the prior and likelihood distribution in the Bayesian decision analysis (BDA), respectively. Finally, the resultant posterior distributions were used to assess the long-term exposure and health-risk posed to fastener manufacturing industry workers due to PAH exposures.

## 2. Material and Methods 

### 2.1. Predicting and Confirming Polycyclic Aromatic Hydrocarbon (PAH) Exposure Concentrations 

#### 2.1.1. Predicting Oil Mist Exposure Concentrations

In the fastener manufacturing industries, threading workers were found to have the highest oil mist exposures [22] and hence were selected in the present study. In our previous study, the fastener production rate (*Pr*; ton/day) was used as a surrogate for predicting oil mist exposure concentrations *(C_p-oil_*; mg/m^3^) [21]. A good prediction model was obtained by the expression:

*C_p-oil_* = 1.42 *Pr* + 0.267 (*R*^2^ = 0.92, *n* = 12, MSE = 0.09) (1)

#### 2.1.2. Predicting Concentrations of Polycyclic Aromatic Hydrocarbons (PAHs) Contained in Metal Work Fluids (MWFs) during One Recycling Period

For the selected threading process, beside the part of the MWFs being emitted to the workplace atmosphere, the rest was continuously recycled from the process and stored in a recycling tank. Friction heat was involved in the threading process which would resulting in either a decrease (due to the evaporation process) or increase in its PAH contents (due to the synthesis process). As a result, concentrations of PAHs contained in MWF could be continuously changing during one recycling cycle. Through our field observations, we found that one recycling cycle started with the recycling MWF tank filled with ~80 kg of new MWFs (viscosity = 6.57 cSt at 40 °C) and ended with ~20 kg of used MWFs (viscosity 82.92 cSt at 40 °C after ~60 days). In the present study, the cumulative fastener production rates (*CPr*) were recorded continuously for one recycling period. A total of 18 MWF samples were collected from the tank on 18 separate days during the cycle.

For each collected sample, ~200 µL MWFs were placed in a solvent solution (a mixture of *n*-hexane and dichloromethane, *v:v* = 1:1, respectively), and extracted in a Soxhlet extractor to perform a 24 h PAH analyses. The extract was then concentrated, cleaned-up, and re-concentrated to exactly 1.0 mL or 0.5 mL. PAH content was determined by using a gas chromatograph (GC; Hewlett-Packard 5890A) with a mass selective detector (MSD; Hewlett-Packard 5972) and a computer workstation. The GC/MS was equipped with a Hewlett-Packard capillary column (HP Ultra 2—50 m × 0.32 mm × 0.17 μm), HP-7673A automatic sampler, injection volume 1 μL, splitless injection at 310 °C, ion source temperature at 310 °C, oven temperature from 50 °C to 100 °C at 20 °C/min; 100 °C to 290 °C at 3 °C/min; and hold at 290 °C for 40 min. The masses of primary and secondary ions of PAHs were determined using the scan mode for pure PAH standards. Qualification of PAHs was performed using the selected ion monitoring (SIM) mode [23,24,25,26,27,28,29,30,31].

The concentrations of 22 PAH compounds were determined, including naphthalene (NaP), acenaphthylene (AcPy), acenaphthene (AcP), fluorene (Flu), phenanthrene (PA), anthracene (Ant), fluoranthene (FL), pyrene (Pyr), cyclopenta[c,d]pyrene (CYC), benz[a]anthracene (BaA), chrysene (CHR), benzo[b]fluoranthene (BbF), benzo[k]fluoranthene (BkF), benz[e]pyrene (BeP), benzo[a]pyrene (BaP), berylene (PER), indeno[1,2,3-cd]pyrene (IND), dibenz[a,h]anthracene (DBA), benzo[b]chrycene (BbC), benzo[ghi]perylene (BghiP), coronene (COR), and dibenzo[a,e]pyrene (DBP). Analysis of the serial dilution of PAH standards show that the limit of detection (LOD) of GC/MS was 0.095ng–1.54 ng.

In this study, the concentration of total PAHs was defined as the sum of the concentrations of the selected 22 PAH compounds. In order for the results of the present study to be comparable with other research data [25,28,31,32], PAH contents were further classified into three categories according to their molecular weights: low molecular weight-PAHs (LMW-PAHs containing two- and three-ringed PAHs), middle molecular weight-PAHs (MMW-PAHs containing four-ringed PAHs), and high molecular weight-PAHs (HMW-PAHs containing five-, six- and seven-ringed PAHs). Furthermore, regression analyses (using *CPr* as a predictor) were conducted to predict total-, LMW-, and HMW-PAHs contained in MWFs (*i.e.*, *C_MWF-Total-PAHs_*, *C_MWF-LMW-PAHs_*, *C_MWF-MMW-PAHs_*, and *C_MWF-HMW-PAHs_*).

#### 2.1.3. Predicting Long Term Exposure Concentrations of Polycyclic Aromatic Hydrocarbons (PAHs)

*Pr* records were collected from the selected industry for one year, and were further converted to *CPr* after matching with the MWF recycling period. Here, *Pr* was used to predict *C_p-oil_*, and the corresponding *CPr* was used to predict *C_MWF-Total-PAHs_*, *C_MWF-LMW-PAHs_*, *C_MWF-MMW-PAHs_*, and *C_MWF-HMW-PAHs_*, respectively. Finally, exposure concentrations of the total-, LMW-, MMW-, and HMW-PAHs (*i.e.*, *C_p-Total-PAHs_*, *C_p-LMW-PAHs_*, *C_p-MMW-PAHs_*, and *C_p-HMW-PAHs_*) can be obtained as follows:

*C_p-Total-PAHs_* = *C_p-oil _*× *C_MWF-Total-PAHs_* (2)

*C_p-LMW-PAHs_* = *C_p-oil _*× *C_MWF-LMW-PAHs_* (3)

*C_p-MMW-PAHs_* = *C_p-oil _*× *C_MWF-MMW-PAHs_* (4)

*C_p-HMW-PAHs_* = *C_oil _*× *C_MWF-HMW-PAHs_* (5)

#### 2.1.4. Confirming Predicted Polycyclic Aromatic Hydrocarbon (PAH) Exposure Concentrations 

To measure personal exposures, a rotating mannequin mounted with a personal sampling train was used and placed beside one randomly selected operator to simulate the worker’s exposure scenario (*i.e.*, the orientation-averaged condition) for collecting samples. The use of the above approach was simply for reducing the interference of manufacturing processes. Samplings were conducted once per month for one year, and a total of 12 samples were collected. The adopted sampling method was modified from the NIOSH method 5515. The sampling train consisted of a filter cassette (IOM personal inhalable aerosol sampler, Catalog No. 225-70, SKC Inc., Eighty-four, PA, USA) and followed by a sorbent tube (polyurethane foam (denoted PUF) plug/3.5 g Amberlite (tm) XAD-2 (denoted as XAD-2) resin/PUF separation layer/0.5 g XAD-2 resin/ PUF plug). The sampling flow rate was 2.0 L/min. Before sampling, all filters and sorbent tubes were cleaned and extracted with a solvent solution (mixture of *n*-hexane and dichloromethane, *v:v* = 1:1) for 24 h in a Soxhlet extractor. After sampling, all filters and sorbent tubes were sent for PAHs analysis to determine the concentrations of both particle phase PAHs and gas phase PAHs. The pretreatment and analysis procedures were similar to those described in the previous section. Five internal standards (Nap-d8, Acp-d10, PA-d10, CHR-d12, and PER-d12) were used to check the response factors and recovery efficiencies for PAHs analysis. The recovery efficiencies of 22 individual PAHs and these five internal standards were determined by processing solutions containing known PAH concentrations through the same experimental procedure as the analyzing samples. The recovery efficiency of PAHs varied between 0.786 and 0.935, with an average of 0.865. The above values were used to adjust the observed concentration. The mean relative standard deviation (RSD) (%) of recovery efficiencies was 5.13% (range 1.28%–8.89%). The recovery efficiencies of five internal standards were between 0.791 and 0.986 and were fairly consistent. The blank tests for PAHs were accomplished by the same procedure as the recovery-efficiency tests without adding known standard solutions before extraction. Analysis of field blanks, including filters and PUF/resin cartridges, showed no significant contaminant. After sample analyses, the measured exposure concentrations of the total-, LMW-, MMW-, and HMW-PAHs (*i.e.*, *C_m-Total-PAHs_*, *C_m-LMW-PAHs_*, *C_m-MMW-PAHs_*, and *C_m-HMW-PAHs_*) were calculated.

For confirmation purposes, twelve *Pr* and *CPr* records corresponding to the sampling days were identified. Then, *C_p-Total-PAHs_*, *C_p-LMW-PAHs_*, *C_p-MMW-PAHs_* and *C_p-HMW-PAHs_* can be obtained via the use of Equations (2–5). Finally the above results were compared with the corresponding *C_m-Total-PAHs_*, *C_m-LMW-PAHs_*, *C_m-MMW-PAHs_*, and *C_m-HMW-PAHs_* for confirmation purposes.

### 2.2. Conducting Long-Term Exposure and Health-Risk Assessments due to Polycyclic Aromatic Hydrocarbons (PAHs) Exposures

#### 2.2.1. Selection Criteria for Conducting Polycyclic Aromatic Hydrocarbons (PAHs) Exposure and Health-Risk Assessments

For exposure assessment, an 8-h time-weighted-average permissible exposure limit (PEL-TWA) of 0.2 mg/m^3^ for total-PAHs promulgated by Taiwan’s government was adopted in the present study. The exposure ratings were classified into five categories of ER0 (*C_Total-PAHs _≤* 0.001 PEL), ER1 (0.001 PEL < *C_Total-PAHs_* *≤* 0.35 PEL), ER2 (0.35 PEL < *C_Total-PAHs _≤* 0.5 PEL), ER3 (0.5 PEL < *C_Total-PAHs _≤* 1.0 PEL), and ER4 (*C_Total-PAHs_* > 1.0 PEL), respectively. Each category can be assigned to a SEG whenever the true 95th percentile exposure falls within the specified range. 

To assess health risks associated with PAH exposures, it is important to know the total carcinogenic potential arising from the exposures to various PAH compounds. In principle, the carcinogenic potency of a given PAH compound can be assessed according to its benzo[a]pyrene equivalent concentration (BaP_eq_). Calculating the BaP_eq_ concentration for a given PAH compound requires the use of its toxic equivalent factor (TEF, using benzo[a]pyrene as a reference compound) to adjust its original concentration. Among the available TEFs lists, the one established by Nisbet and LaGoy in 1992 has been demonstrated to best reflect the toxic potency of each individual PAH species [33]. For those lacking TEFs (CYC, BeP, PER, COR, and DBP), values suggested by other researchers were adopted in this study [34,35]. Table 1 show the TEF list used in the present study. The carcinogenic potency of total PAHs (*C_total-BaPeq_*) can be determined as the sum of BaP_eq_ concentrations of the 22 selected PAH compounds. 

**Table 1:** Polycyclic Aromatic Hydrocarbon (PAH) compounds and their toxic equivalent factors (TEFs).

Polycyclic Aromatic Hydrocarbon (PAH)	TEFs Used in This Study
Naphthalene (Nap)	0.001
Acenaphthylene (AcPy)	0.001
Acenaphthene (Acp)	0.001
Fluorene (Flu)	0.001
Phenanthrene (PA)	0.001
Anthracene (Ant)	0.01
Fluoranthene (FL)	0.001
Pyrene (Pyr)	0.001
Cyclopenta(c,d)pyrene (CYC)	0.1
Benzo(a)anthracene (BaA)	0.1
Chrysene (CHR)	0.01
Benzo(b)fluoranthene (BbF)	0.1
Benzo(k)fluoranthene (BkF)	0.1
Benzo(e)pyrene (BeP)	0.01
Benzo(a)pyrene (BaP)	1
Perylene (PER)	0.001
Indeno(1,2,3,-cd)pyrene (IND)	0.1
Dibenzo(a,h)anthracene (DBA)	1
Benzo(b)chrycene (BbC)	-- *
Benzo(ghi)perylene (BghiP)	0.01
Coronene (COR)	0.001
Dibenzo(a,e)pyrene (DBP)	1

* no TEF value is available.

For estimating the excess lung cancer risk associated with inhalation PAH exposures, the World Health Organization (WHO) has suggested a unit risk of 8.7 × 10^−2^ (µg/m^3^)^−1^ for the lifetime (=70 years) PAH exposure, assuming BaP exposure concentration of 1 µg/m^3 ^ [36]. It is worth noting that the above unit risk was proposed for lifetime exposure, therefore, it has been adopted for assessing the exposure of general adults to the ambient atmospheric PAHs [37]. For occupational exposure, Pott established a relationship between BaP exposure and lung cancer risk based on an epidemiological database [38]. Pott suggested the unit risk of 7.0 × 10^−2^ (µg/m^3^)^−1^ for a 25-year occupational PAHs exposure with the averaged BaP concentration of 1 µg/m^3^. Despite using the same data bank, the United States Environmental Protection Agency suggested a different unit risk of 6.4 × 10^−4^ (µg/m^3^)^−1^ for PAHs exposure based on total PAHs (expressed as the benzene soluble fractions). Since a recent study has indicated BaP could be a better indicator than total PAHs for characterizing the carcinogenic potency of PAHs, the unit risk suggested by Pott in 1985 was used in our previous study [31] and the present study. Here, the excessive lung cancer risk can be expressed as follows:

Lung Cancer Risk (LCR) = C*_total-BaPeq _*×7.0 × 10^−2^ (6)

Regarding the acceptable excessive cancer risk, the values of 10^−3^, defined as the significant risk level by the US Supreme Court in 1980, was adopted in this study. The cancer risks (CRs) were classified into five categories as CR0 (*LCR* ≤ 2.5 × 10^−3^), CR1 (2.5 × 10^−3^ < *LCR* ≤ 5 × 10^−3^), CR2 (5 × 10^−3^ < *LCR* ≤ 37.5 × 10^−3^), CR3 (37.5 × 10^−3^ <* LCR* ≤ 50 × 10^−3^), and CR4 (*LCR* > 50 × 10^−3^), respectively.

Indeed, MWFs have many other constituents which might pose health-risks to fastener manufacturing workers. Due to both cost and manpower constraints, this study simply focused on conducting long-term exposure and health-risk assessments associated with PAH exposures.

#### 2.2.2. Using the BDA to Conduct Long-Term Exposure and Health-Risk Assessments

In this study, the year-long *C_p-Total-PAHs_* were used to determine the predicted excess lung cancer risk (*LCRp*) posed to workers. On the other hand, the twelve measured total-PAHs exposure concentrations (*i.e*., *C_m-Total-PAHs_*) were used to determine the measured excessive lung cancer risks (*i.e*., *LCRm*). The software of the IH Data AnalystV1.27 (Exposure Assessment Solutions, Inc., Morgantown, WV, USA) was used to conduct the BDA. The BDA software assumes that all data sets can be best described by a single lognormal distribution which is in accordance with the obtained *C_p-Total-PAHs_* and *C_m-Total-PAHs_*. Here*,* the prior (*i.e.*, *C_p-Total-PAHs_* or *LCRp*) represents a priori probability distribution that the 95th percentile of predicted exposure (or risk) falls into each of the five exposure (or risk) rating categories. Similarly, the likelihood distribution describes the probability of the 95th percentile of measured exposure (or risk) (*i.e*., *C_m-Total-PAHs_* or *LCRm*) at each exposure (or risk) rating categories. Finally, the resultant posterior distribution of the 95th percentile of the exposure (or risk) across the five exposure rating categories was determined with 90% confidence. 

## 3. Results and Discussion

### 3.1. Predicting and Confirming Exposure Concentrations of Polycyclic Aromatic Hydrocarbons (PAHs) 

#### 3.1.1. Polycyclic Aromatic Hydrocarbons (PAHs) Contained in Metal Work Fluids (MWFs) during One Recycling Period

Table 2 shows concentrations of the 22 analyzed PAH compounds, LMW-PAHs, MMW-PAHs, HMW-PAHs, total PAHs, and total BaPeq of the 18 collected MWF samples. It can be seen that LMW-, MMW-, and HMW-PAHs account for 97.2% (=4.51 × 10^7^ ng/g), 2.26% (=1.05 × 10^6^ ng/g), and 0.54% (3.14 × 10^5 ^ng/g) of the total-PAHs (=4.64 × 10^7 ^ng/g), respectively. Obviously, total PAHs in MWFs were dominated by LMW-PAHs. The above results were consistent with our previous findings [32].

**Table 2:** Concentrations of the 22 analyzed PAH compounds, LMW-PAHs, MMW-PAHs, HMW-PAHs, total PAHs and total BaPeq in Metal Work Fluids (MWFs) (*n* = 18, units: ng/g).

PAH Compound	Mean	Min.	Max.	SD	GSD
NaP	3.45 × 10^6^	1.11 × 10^6^	9.21 × 10^6^	2.51 × 10^6^	1.95
AcPY	2.16 × 10^6^	5.80 × 10^5^	4.01 × 10^6^	1.01 × 10^6^	1.73
AcP	7.83 × 10^5^	2.37 × 10^5^	1.66 × 10^6^	4.14 × 10^5^	1.76
Flu	6.12 × 10^6^	2.45 × 10^6^	1.30 × 10^7^	2.81 × 10^6^	1.58
PA	1.60 × 10^7^	5.45 × 10^6^	3.72 × 10^7^	8.59 × 10^6^	1.71
Ant	1.65 × 10^7^	5.73 × 10^6^	3.98 × 10^7^	8.96 × 10^6^	1.71
FL	1.59 × 10^5^	5.12 × 10^4^	3.64 × 10^5^	8.36 × 10^4^	1.73
Pyr	5.00 × 10^5^	2.18 × 10^5^	8.32 × 10^5^	1.54 × 10^5^	1.39
BaA	9.64 × 10^4^	5.31 × 10^4^	1.75 × 10^5^	3.33 × 10^4^	1.40
CHR	2.97 × 10^5^	5.85 × 10^4^	5.31 × 10^5^	1.42 × 10^5^	1.80
CYC	9.74 ×10^4^	5.54×10^4^	1.45 × 10^5^	2.39 × 10^4^	1.29
BbF	1.28 × 10^4^	7.02×10^3^	1.62 × 10^4^	2.33 × 10^3^	1.23
BkF	2.05 × 10^4^	1.09 × 10^4^	2.43 × 10^4^	3.52 × 10^3^	1.23
BeP	1.54 × 10^4^	8.49 × 10^3^	5.47 × 10^4^	1.34 × 10^4^	1.72
BaP	1.07 × 10^5^	3.01 × 10^4^	1.37 × 10^5^	2.98 × 10^4^	1.50
Per	2.27 × 10^4^	4.47 × 10^3^	3.04 × 10^4^	6.89 × 10^3^	1.65
IND	3.82 × 10^4^	1.17 × 10^4^	6.11 × 10^4^	1.27 × 10^4^	1.55
DBA	ND	--	--	--	--
BbC	ND	--	--	--	--
BghiP	ND	--	--	--	--
COR	ND	--	--	--	--
DBP	ND	--	--	--	--
LMW-PAHs	4.51 × 10^7^	1.56 × 10^7^	1.05 × 10^8^	2.41 × 10^7^	1.71
MMW-PAHs	1.05 × 10^6^	4.77 × 10^5^	1.71 × 10^6^	3.66 × 10^5^	1.45
HMW-PAHs	3.14 × 10^5^	2.09 × 10^5^	3.61 × 10^5^	3.91 × 10^4^	1.15
Total PAHs	4.64 × 10^7^	1.63 × 10^7^	1.07 × 10^8^	2.45 × 10^7^	1.69
Total BaPeq	3.31 × 10^5^	2.15 × 10^5^	5.32 × 10^5^	8.37 × 10^4^	1.28

#### 3.1.2. Predicting Concentrations of Polycyclic Aromatic Hydrocarbons (PAHs) Containing in Metal Work Fluids (MWFs)

**Figure 1:**
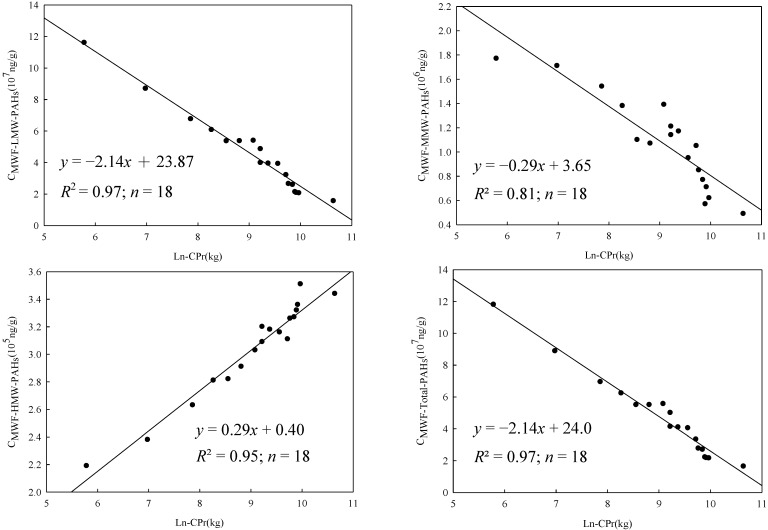
The relationship between CPr (kg/day) and C_MWF-Total-PAHs_, C_MWF-LMW-PAHs_. C_MWF-MMW-PAHs_, and C_MWF-HMW-PAHs_ (*n* = 18).

In the present study, 18 *CPr* values were used to establish models for predicting the concentrations of PAHs contained in MWFs (*i.e.*, *C_MWF-Total-PAHs_*, *C_MWF-LMW-PAHs_*, *C_MWF-MMW-PAHs_*, and *C_MWF-HMW-PAHs_*). Figure 1 shows the relationship between *CPr* (kg/day) and *C_MWF-Total-PAHs_*/*C_MWF-LMW-PAHs_*/*C_MWF-MMW-PAHs_*/*C_MWF-HMW-PAHs_*. Results show that the former three decreases as Ln*CPr* increases, but a totally different trend was found for *C_MWF-HMW-PAHs_*. Simple linear regression analyses yields:

*C_MWF-Total-PAHs_* = −2.16Ln*CPr* + 24.2, *R*^2 ^= 0.97, F = 528.5, *p* < 0.05 (7)

*C_MWF-LMW-PAHs_* = −2.14Ln*CPr* + 23.9, *R*^2 ^= 0.97, F = 532.5, *p* < 0.05 (8)

*C_MWF-MMW-PAHs_* = −0.29Ln*CPr* + 3.66, *R*^2 ^= 0.81, F = 68.7, *p* < 0.05 (9)

*C_MWF-HMW-PAHs_* = 0.29Ln*CPr* + 0.40, *R*^2 ^= 0.95, F = 304.7, *p* < 0.05 (10)

Equations (7‒10) all meet assumptions of linear regression, diagnosed by using SPSS Statistics version 20. The Levene test shows that equations (7‒10) have equal variances (*p* > 0.05). Besides, ANOVA results indicate Equations (7‒10) have a significant relationship between *CPr* and *C_MWF-Total-PAHs_*, *C_MWF-LMW-PAHs_*, *C_MWF-MMW-PAHs_*, and *C_MWF-HMW-PAHs _*(*p* < 0.05). The above high (*R*^2^ = 0.81–0.97) indicates that *CPr* was a suitable surrogate for predicting PAH concentrations in MWFs at different stage. However, it should be noted that Equations (7‒10) are applicable only when LnCPr falls between 5 kg and 11 kg.

Negative regression coefficients were obtained for both predictive models of *C_MWF-LMW-PAHs_* (= −2.14) and *C_MWF-MMW-PAHs _*(= −0.29). The above results were theoretically plausible because higher *CPr* would results in more evaporation of PAH compounds into the workplace atmosphere. Considering MMW-PAHs were less volatile than LMW-PAHs, it is not so surprising to see that the slope (*i.e.*, the regression coefficient) for predicting *C_MWF-LMW-PAHs_* (= −2.14) was steeper than that of *C_MWF-MMW-PAHs_* (= −0.29). Furthermore, a positive regression coefficient (= 0.29) was found in the regression equation for predicting *C_MWF-HMW-PAHs_* that requires further discussion. It is known that HMW-PAHs have inherently low vapor pressures, and hence low evaporation rates can be expected. On the other hand, an increase in *CPr* would also result in the loss in MWFs’ volume (due to the evaporation of highly volatile fractions) which might lead to the increase in *C_MWF-HMW-PAHs_*. Finally, since total-PAHs were dominated mostly by LMW-PAHs (accounting for 97.2% of the total-PAHs), the regression coefficient for predicting *C_MWF-Total-PAHs_* (= −2.16) was close to that of *C_MWF-LMW-PAHs_* (= −2.14).

#### 3.1.3. Polycyclic Aromatic Hydrocarbon (PAH) Exposure Concentrations

Table 3 shows that gas phase and particle phase total PAHs account for 99.0% (=1.25 × 10^5^ ng/m^3^) and 1.00% (=1.27 × 10^3^ ng/m^3^) of total PAH concentrations (*i.e*., gas + particle phase = 1.26 × 10^5^ ng/m^3^), respectively. By examining PAH homologue distributions for both gas and particle phase PAHs, it can be seen that the fractions of LMW-, MMW-, and HMW-PAHs for gas phase were 98.6%, 1.4%, and 0%, and for particle phase they were 63.2%, 18.1%, and 18.7%, respectively. Gas phase PAHs had a higher fraction in LMW-PAHs, which could be due to their intrinsically high volatility. On the contrary, a higher fraction in both MMW-PAHs and HMW-PAHs was found in the particle phase PAHs, which could be due to their lower volatility. 

For total BaPeq concentrations, the gas phase (=1.41 × 10^2^ ng/m^3^) was higher than that of particle phase (=95.7 ng/m^3^) accounting for 59.6% and 40.4% of the total (=2.37 × 10^2^ ng/m^3^), respectively. Because gas phase PAHs were dominated by PAHs with lower TEFs (*i.e.*, LMW-PAHs; see Table 1), and particle phase PAHs were dominated by PAHs with higher TEFs (*i.e.*, MMW-PAHs and HMW-PAHs; see Table 1), the results obtained from the present study could be theoretically plausible.

#### 3.1.4. Confirmation of the Proposed Predicting Models

**Figure 2:**
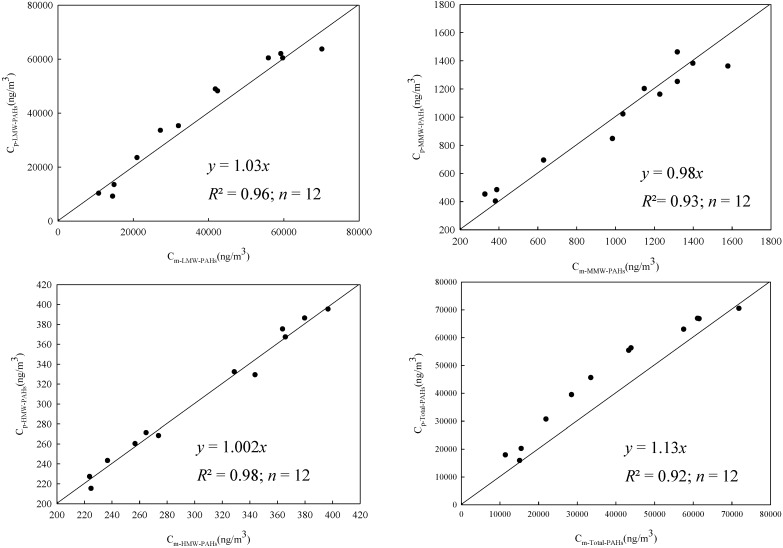
Comparing measured Polycyclic Aromatic Hydrocarbon (PAH) concentrations with the corresponding predicted Polycyclic Aromatic Hydrocarbon (PAH) concentrations.

In this study, all *Pr* records and their corresponding *CPr* were obtained from industry records for the selected year. For the confirmation purpose, 12 *Pr* and *CPr* records corresponding to mannequin samplings days were identified. Here, the former was used to predict *C_oil-p_* (based on Equation (1)), and the latter was used to predict *C_MWF-Total-PAHs_*, *C_MWF-LMW-PAHs_*, *C_MWF-MMW-PAHs_*, and *C_MWF-HMW-PAH_* (based on Equations (7–10)). Then, *C_p-Total-PAHs_*, *C_p-LMW-PAHs_*, *C_p-MMW-PAHs_*, and *C_p-HMW-PAHs_* can be obtained by applying the above results to Equations (2–5). Finally the above results were compared with the corresponding *C_m-Total-PAHs_*, *C_m-LMW-PAHs_*, *C_m-MMW-PAHs_* , and *C_m-HMW-PAHs_* for the confirmation purposes. Figure 2 shows the relationship between the measured PAH concentrations (*i.e.*, *C_m-Total-PAHs_*, *C_m-LMW-PAHs_*, *C_m-MMW-PAHs_* , and *C_m-HMW-PAHs_*) and the corresponding predicted PAH concentrations (*i.e.*, *C_p-Total-PAHs_*, *C_p-LMW-PAHs_*, *C_p-MMW-PAHs_*, and *C_p-HMW-PAHs_*). This study yielded regression results as followings:

*C_p-Total-PAHs_* = 1.13 × *C_m-Total-PAHs_* (*R*^2 ^= 0.92, F = 826.2, *p* < 0.05) (11)

*C_p-LMW-PAHs_* = 1.03 × *C_m-LMW-PAHs_* (*R*^2 ^= 0.96, F = 1187, *p* < 0.05) (12)

*C_p-MMW-PAHs_* = 0.98 × *C_m-MMW-PAHs_* (*R*^2 ^= 0.93, F = 1199, *p* < 0.05) (13)

*C_p-HMW-PAHs_* = 1.00 × *C_m-HMW-PAHs_* (*R*^2 ^= 0.99, F = 20798, *p* < 0.05) (14)

Since all obtained regression equations were statistically significant (*p* < 0.05) with high *R*^2^ (= 0.92–0.99), and their regression coefficient were consistently close to unity (=0.98–1.13) indicating that the proposed integrated approach was adequate for predicting PAH exposure concentrations.

**Table 3:** Measured exposure concentrations of 22 selected Polycyclic Aromatic Hydrocarbon (PAH) compounds, LMW-PAHs, MMW-PAHs, HMW-PAHs, total PAHs and total BaPeq (*n* = 12) (unit: ng/m^3^).

PAH Compounds	Gas Phase				Particle Phase				G as phase + Particle Phase			
	Mean	Min.	Max.	SD.	Mean	Min.	Max.	SD.	Mean	Min.	Max.	SD.
NaP	1.13 × 10^5^	1.36 × 10^4^	4.66 × 10^5^	1.63 × 10^5^	5.35 × 10^2^	5.03 × 10^1^	9.87 × 10^2^	3.58 × 10^2^	1.13 × 10^5^	1.38 × 10^4^	4.67 × 10^5^	1.64 × 10^5^
AcPY	7.42 × 10^2^	4.43 × 10^2^	1.75 × 10^3^	3.44 × 10^2^	3.19 × 10^1^	2.24 × 10^1^	4.00 × 10^1^	7.33 × 10°	7.74 × 10^2^	4.65 × 10^2^	1.77 × 10^3^	3.42 × 10^2^
AcP	1.23 × 10^3^	5.09 × 10^2^	2.27 × 10^3^	7.66 × 10^2^	2.56 × 10^1^	1.18 × 10^1^	3.84 × 10^1^	1.29 × 10^1^	1.26 × 10^3^	5.47 × 10^2^	2.28 × 10^3^	7.54 × 10^2^
Flu	4.05 × 10^3^	8.70 × 10^2^	1.02 × 10^4^	3.50 × 10^3^	4.74 × 10^1^	3.12 × 10^1^	5.75 × 10^1^	6.69	4.10 × 10^3^	9.15 × 10^2^	1.02 × 10^4^	3.50 × 10^3^
PA	3.80 × 10^3^	8.56 × 10^2^	9.07 × 10^3^	3.13 × 10^3^	1.33 × 10^2^	5.01 × 10^1^	2.43 × 10^2^	8.32 × 10^1^	3.93E+03	9.11 × 10^2^	9.31 × 10^3^	3.21 × 10^3^
Ant	3.73 × 10^2^	2.73 × 10^1^	5.19 × 10^2^	1.22 × 10^2^	2.77 × 10^1^	1.04 × 10^1^	4.20 × 10^1^	1.31 × 10^1^	4.01 × 10^2^	4.17 × 10^1^	5.38 × 10^2^	1.28 × 10^2^
FL	8.07 × 10^2^	4.26 × 10^2^	1.36 × 10^3^	3.86 × 10^2^	6.49 × 10^1^	1.31 × 10^1^	1.22 × 10^2^	2.89 × 10^1^	8.72 × 10^2^	4.74 × 10^2^	1.44 × 10^3^	3.99 × 10^2^
Pyr	6.64 × 10^2^	3.62 × 10^2^	1.06 × 10^3^	3.00 × 10^2^	7.09 × 10^1^	4.39 × 10^1^	1.16 × 10^2^	2.80 × 10^1^	7.35 × 10^2^	4.07 × 10^2^	1.13 × 10^3^	3.20 × 10^2^
BaA	1.14 × 10^2^	ND	2.35 × 10^2^	1.19 × 10^2^	4.97 × 10^1^	ND	7.21 × 10^1^	1.72 × 10^1^	1.64 × 10^2^	4.80 × 10^1^	2.96 × 10^2^	1.19 × 10^2^
CHR	1.83 × 10^2^	ND	4.64 × 10^2^	2.00 × 10^2^	4.37 × 10^1^	ND	5.49 × 10^1^	1.47 × 10^1^	2.27 × 10^2^	4.64 × 10^1^	5.10 × 10^2^	1.93 × 10^2^
CYC	ND	ND	ND	ND	2.19 × 10^1^	1.05 × 10^1^	3.54 × 10^1^	9.18	2.19 × 10^1^	1.05 × 10^1^	3.54 × 10^1^	9.18
BbF	ND	ND	ND	ND	4.15 × 10^1^	3.56 × 10^1^	4.49 × 10^1^	2.69	4.15 × 10^1^	3.56 × 10^1^	4.49 × 10^1^	2.69
BkF	ND	ND	ND	ND	2.76 × 10^1^	1.14 × 10^1^	4.41 × 10^1^	1.45 × 10^1^	2.76 × 10^1^	1.14 × 10^1^	4.41 × 10^1^	1.45 × 10^1^
BeP	ND	ND	ND	ND	2.13 × 10^1^	ND	6.28 × 10^1^	2.36 × 10^1^	2.13 × 10^1^	ND	6.28 × 10^1^	2.36 × 10^1^
BaP	ND	ND	ND	ND	7.69 × 10^1^	4.40 × 10^1^	1.28 × 10^2^	3.47 × 10^1^	7.69 × 10^1^	4.40 × 10^1^	1.28 × 10^2^	3.47 × 10^1^
per	ND	ND	ND	ND	1.81 × 10^1^	3.47	2.80 × 10^1^	1.00 × 10^1^	1.81 × 10^1^	3.47	2.80 × 10^1^	1.00 × 10^1^
IND	ND	ND	ND	ND	2.83 × 10^1^	2.83 × 10^1^	2.83 × 10^1^	ND	2.83 × 10^1^	2.83 × 10^1^	2.83 × 10^1^	ND
DBA	ND	ND	ND	ND	ND	ND	ND	ND	ND	ND	ND	ND
BbC	ND	ND	ND	ND	ND	ND	ND	ND	ND	ND	ND	ND
BghiP	ND	ND	ND	ND	ND	ND	ND	ND	ND	ND	ND	ND
COR	ND	ND	ND	ND	ND	ND	ND	ND	ND	ND	ND	ND
DBP	ND	ND	ND	ND	ND	ND	ND	ND	ND	ND	ND	ND
LMW-PAHs	1.23 × 10^5^	1.69 × 10^4^	4.76 × 10^5^	1.67 × 10^5^	8.00 × 10^2^	8.00 × 10^2^	2.76 × 10^2^	1.31 × 10^3^	1.24 × 10^5^	1.73 × 10^4^	4.77 × 10^5^	1.67 × 10^5^
MMW-PAHs	1.77 × 10^3^	7.88 × 10^2^	3.07 × 10^3^	9.95 × 10^2^	2.29 × 10^2^	2.29 × 10^2^	1.44 × 10^2^	3.41 × 10^2^	2.00 × 10^3^	9.86 × 10^2^	3.26 × 10^3^	1.02 × 10^3^
HMW-PAHs	ND	ND	ND	ND	2.36 × 10^2^	2.36 × 10^2^	1.94 × 10^2^	2.75 × 10^2^	2.36 × 10^2^	1.94 × 10^2^	2.75 × 10^2^	2.39 × 10^1^
Total PAHs	1.25 × 10^5^	1.77 × 10^4^	4.79 × 10^5^	1.68 × 10^5^	1.27 × 10^3^	1.27 × 10^3^	7.03 × 10^2^	1.78 × 10^3^	1.26 × 10^5^	1.86 × 10^4^	4.80 × 10^5^	1.68 × 10^5^
Total BaPeq	1.41 × 10^2^	2.14E+01	5.06 × 10^2^	1.76 × 10^2^	9.57 × 10^1^	9.57 × 10^1^	6.45 × 10^1^	1.47 × 10^2^	2.37 × 10^2^	8.59 × 10^1^	6.44 × 10^2^	1.99 × 10^2^

### 3.2. Long-Term Polycyclic Aromatic Hydrocarbon (PAH) Exposure and Health Risk Assessment for Threading Workers

#### 3.2.1. Long-Term Polycyclic Aromatic Hydrocarbon (PAH) Exposure Assessment

**Figure 3:**
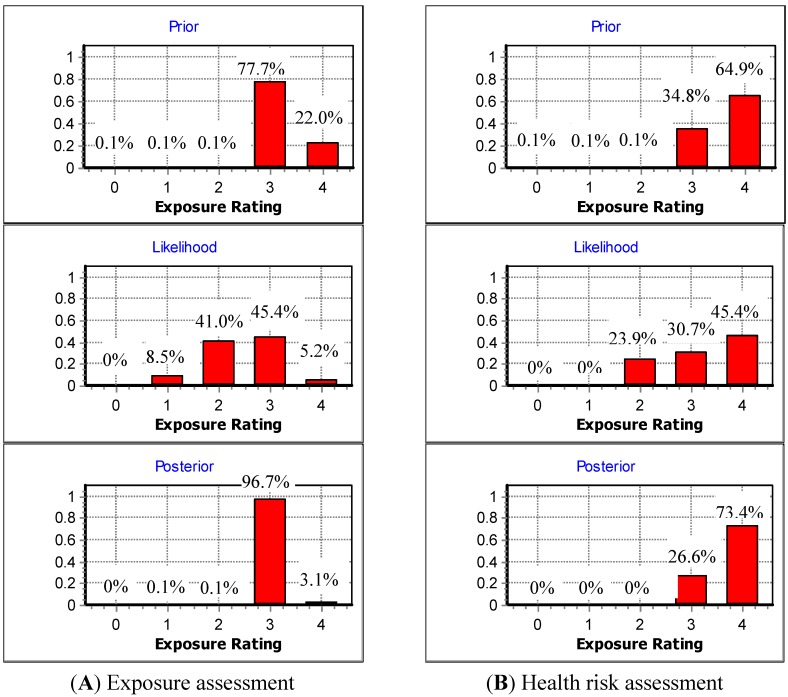
The resultant prior, likelihood, and posterior distributions for (**A**) long-term Polycyclic Aromatic Hydrocarbon (PAH) exposure assessment; and (**B**) health risk assessment.

PAH exposure ratings (ER) were classified into five categories of ER0(*C_Total-PAHs _*≤ 0.001PEL), ER1 (0.001PEL < *C_Total-PAHs _*≤ 0.35PEL), ER2 (0.35PEL < *C_Total-PAHs _*≤ 0.5PEL), ER3 (0.5PEL < *C_Total-PAHs_* ≤ 1.0PEL), and ER4 = (*C_Total-PAHs_* > 1.0PEL), respectively. Parameter spaces were defined as GM = 4.08 × 10^−6^−1, and GSD = 1.05−4, respectively. In this study, the year-long *C_p-Total-PAHs_*, and the twelve *C_m-Total-PAHs_* served as the prior and likelihood distribution in BDA, respectively. The resultant posterior distribution was used to assess workers’ long term PAH exposures. Figure 3(A) shows the prior, likelihood, and posterior distributions obtained from the present study. For the prior distribution, PAH exposures mainly fell to the two categories of the ER3 (=77.7%) and ER4 (=22.0%). The above results were somewhat different from that of the likelihood distribution (ER2=41.0% and ER3= 45.4%). The above results suggest that simply using limited measured data might not be adequate for assessing long-term exposures. In particular, in this case the use of limited measured data might result in the underestimation of workers’ long-term exposures. Finally, the resultant posterior distribution suggests that workers’ PAH long-term exposures, though they only had a 3.1% chance being higher than PEL-TWA, had a 96.7% chance of being above the action level (*i.e.*, 0.5 PEL-TWA). Therefore, measures are encouraged to be taken to reduce PAH emissions from the threading process, such as the installation of local exhaust ventilation, curtains at the oil mist release sites, and providing suitable personal respiratory protective equipment for workers. Here, it should be noted that the results obtained from the posterior distribution can only be regarded as the best estimate based on the currently available prediction and measured data.

#### 3.2.2. Long Term Health Risk Assessment

In this study, *C_p-Total-BapEq_* and *C_m-Total__-BapEq_* served as the prior and the likelihood distribution in conducting the BDA, respectively. The excessive cancer risk ratings (CR) were classified into five categories: CR0 (*LCR* ≤ 2.5 × 10^−3^), CR1 (2.5 × 10^−3^ < *LCR* ≤ 5 × 10^−3^), CR2 (5 × 10^−3^ < *LCR* ≤ 37.5 × 10^−3^), CR3 (37.5 × 10^−3^ < *LCR* ≤ 50 × 10^−3^), and CR4 (LCR > 50 × 10^−3^). Parameter spaces were defined as GM = 5.11 × 10^−5^−0.25, and GSD = 1.05−4, respectively. Figure 3(B) shows the prior, likelihood, and posterior distributions obtained from the present study. For the prior distribution, *LCR* mainly fell to both categories of the *ER3* (=34.8%) and *ER4* (=64.9%). For the likelihood distribution, though *LCR* also mainly fell to both categories of the *ER3* (=30.7%) and *ER4* (=45.4%), there still was a 23.9% probability that fell to the* ER2* category. Therefore, simply using limited measured data might not be feasible for assessing health risks associated with long term PAH exposures. In particular, if only limited measured data were used in this case, an underestimation of the excessive cancer risks might occur. Finally, the resultant posterior distribution suggests that the obtained excessive cancer risks mainly fell to both categories of the *ER3* (=26.6%) and *ER4* (=73.4%). Since there was 73.4% chance to be above the acceptable health risk, appropriate control measures should be taken to reduce the health risk posed to the threading workers. Again, it should be noted that the resultant posterior distribution can only be regarded as the best solution based on the currently available predicted and measured data.

## 4. Conclusions

Though only a small amount of field sampling data were available, this study developed an integrated approach for conducting exposure and health-risk assessments associated with PAH exposure of threading workers in a fastener manufacturing industry. This study found that using both *Pr* and *CPr* as predictors in the two proposed predictive models, long-term PAH exposure concentrations could be effectively determined. Using the predicted long-term PAH exposure concentrations and limited measured data respectively as the prior and likelihood distribution in the BDA, the resultant posterior distributions could more effectively determine the long-term exposure and health-risks posed on workers. This study yielded that there were ～3.1%, 96.7%, and 73.4% chances for threading workers to be above the PEL-TWA (0.2 mg/m^3^), action level (0.1 mg/m^3^), and acceptable health risk of 10^−3^, respectively. Therefore, it is suggested that preventive measures should be taken to reduce workers’ PAH exposures immediately. However, it should be noted that the proposed predictive models are only applicable when *Pr* and *CPr* fell to the ranges designated in the present study. In addition, the resultant posterior distributions can only be regarded as the best solution based on the currently available information. Improvements could be made in the future, such as increasing the sample size of the measured data (*i.e.*, likelihood), or the development of new surrogates for better charactering the prior distribution.
